# Rhinovirus increases *Moraxella catarrhalis* adhesion to the respiratory epithelium

**DOI:** 10.3389/fcimb.2022.1060748

**Published:** 2023-01-17

**Authors:** Eishika Dissanayake, Rebecca A. Brockman-Schneider, Reed M. Stubbendieck, Britney A. Helling, Zhumin Zhang, Yury A. Bochkov, Charmaine Kirkham, Timothy F. Murphy, Carole Ober, Cameron R. Currie, James E. Gern

**Affiliations:** ^1^ Department of Pediatrics, University of Wisconsin – Madison, Madison, WI, United States; ^2^ Department of Bacteriology, University of Wisconsin – Madison, Madison, WI, United States; ^3^ Department of Human Genetics, University of Chicago, Chicago, IL, United States; ^4^ Department of Biostatistics and Medical Informatics, University of Wisconsin – Madison, Madison, WI, United States; ^5^ Clinical and Translational Research Center, Jacobs School of Medicine and Biomedical Sciences, University at Buffalo, The State University of New York, Buffalo, NY, United States; ^6^ Michael G. DeGroote Institute for Infectious Disease Research, David Braley Centre for Antibiotic Discovery, Department of Biochemistry and Biomedical Sciences, McMaster University, Hamilton, ON, Canada

**Keywords:** *Moraxella catarrhalis*, rhinovirus, airway epithelium, co-infection, asthma

## Abstract

Rhinovirus causes many types of respiratory illnesses, ranging from minor colds to exacerbations of asthma. *Moraxella catarrhalis* is an opportunistic pathogen that is increased in abundance during rhinovirus illnesses and asthma exacerbations and is associated with increased severity of illness through mechanisms that are ill-defined. We used a co-infection model of human airway epithelium differentiated at the air-liquid interface to test the hypothesis that rhinovirus infection promotes *M. catarrhalis* adhesion and survival on the respiratory epithelium. Initial experiments showed that infection with *M. catarrhalis* alone did not damage the epithelium or induce cytokine production, but increased trans-epithelial electrical resistance, indicative of increased barrier function. In a co-infection model, infection with the more virulent rhinovirus-A and rhinovirus-C, but not the less virulent rhinovirus-B types, increased cell-associated *M. catarrhalis*. Immunofluorescent staining demonstrated that *M. catarrhalis* adhered to rhinovirus-infected ciliated epithelial cells and infected cells being extruded from the epithelium. Rhinovirus induced pronounced changes in gene expression and secretion of inflammatory cytokines. In contrast, *M. catarrhalis* caused minimal effects and did not enhance RV-induced responses. Our results indicate that rhinovirus-A or C infection increases *M. catarrhalis* survival and cell association while *M. catarrhalis* infection alone does not cause cytopathology or epithelial inflammation. Our findings suggest that rhinovirus and *M. catarrhalis* co-infection could promote epithelial damage and more severe illness by amplifying leukocyte inflammatory responses at the epithelial surface.

## Introduction

Rhinovirus (RV) infections can cause respiratory illnesses ranging from mild colds to lower respiratory tract infections with wheezing. The RV-A and RV-C types are particularly associated with wheezing illnesses and hospitalizations due to asthma exacerbations (Bizzintino et al., 2011; Lee et al., 2012). *Moraxella catarrhalis* is an opportunistic pathogen that can cause otitis media, sinusitis, and exacerbations of chronic obstructive pulmonary disease. *Moraxella catarrhalis* abundance is also increased in nasal secretions of children during RV upper respiratory and wheezing illnesses (Kloepfer et al., 2014; Bashir et al., 2018). RV infections precede increased bacterial detection during these illnesses, and their concurrent or sequential detection is associated with an increased incidence of wheezing illnesses and asthma exacerbations (Kloepfer et al., 2014).

Respiratory viruses can promote secondary bacterial infections by inducing receptors that increase bacterial binding, which is the first step in bacterial invasiveness. In studies using several respiratory viruses, virus infections increased the expression of respiratory epithelial receptors that can be used by *Haemophilus influenzae (* Sajjan et al., 2006; Avadhanula et al., 2006) and *Streptococcus pneumoniae (* Avadhanula et al., 2006). *Moraxella catarrhalis* has several outer membrane proteins (OMPs) (e.g., UspA1, UspA2, and Hemagglutinin [Hag/MID]) that can bind to multiple cellular proteins, including carcinoembryonic antigen-related cell adhesion molecule (CEACAM) and matrix proteins, including fibronectin and laminin (Balder et al., 2009). CEACAM1, CEACAM5 (carcinoembryonic antigen or CEA), and 6 are expressed in the respiratory epithelium in varying co-expression patterns (Klaile et al., 2013). Secreted CEACAM5 and 6 are detected in normal bronchial mucus (Matsuoka et al., 1990; Klaile et al., 2013). CEACAM1 can serve as a receptor for the respiratory pathogens *M. catarrhalis*, non-typeable *Haemophilus influenzae*, and *Neisseria meningitidis* and the urogenital pathogen *N. gonorrhoeae (* Virji et al., 2000; Hill et al., 2005). In addition to bacterial binding, CEACAM engagement also triggers endocytosis and transcytosis of *N. gonorrhoeae* allowing epithelial barrier function to remain intact (Wang et al., 1998). Virus-induced interferon responses may promote respiratory tract colonization by pathobionts, including *M. catarrhalis*, by dampening innate immune responses necessary for bacterial clearance (Sun and Metzger, 2008; de Steenhuijsen Piters et al., 2022). Finally, viral infections can disturb epithelial cell barrier function, and this activity can promote invasiveness. However, the specific mechanisms that cause RV-*M. catarrhalis* co-infections to increase the abundance of bacteria in the airways and the severity of illness are currently unknown.

In this study, we tested the hypothesis that RV infection promotes *M. catarrhalis* adhesion and survival on the respiratory epithelium by increasing the production of *M. catarrhalis* OMPs and upregulating CEACAM production on airway epithelial cells. To test this hypothesis, we used an *in vitro* model of differentiated bronchial epithelial cells and identified effects of RV co-infection on *M. catarrhalis* cell association, abundance, and cellular responses.

## Results

### *Moraxella catarrhalis* adherence and survival on differentiated airway epithelial cells

**Figure 1:**
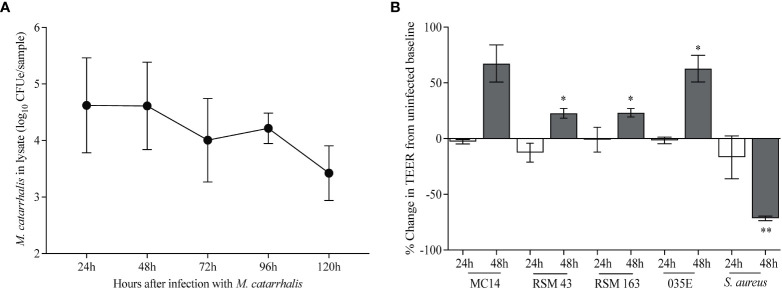
*M. catarrhalis* survival on airway epithelium. Fully differentiated epithelial cell cultures from a single donor were infected with **(A)** *M. catarrhalis* strain MC14, and the infection was allowed to persist over five days. Cell-associated *M. catarrhalis* CFUe was quantified at each timepoint using quantitative PCR. The result is a combination of four independent experiments. Error bars represent geometric mean ± SD. **(B)** Percentage change in TEER at 24 and 48 h following infection with either *M. catarrhalis* MC14, RSM43, RSM163, 035E or a clinical isolate of *S. aureus*. Bars represent the mean ± SEM from 3 independent experiments (^*^ *P* < 0.05, ^**^ *P* < 0.01 vs. uninfected control; paired *t*-test).

To determine the effects of *M. catarrhalis* on airway epithelial cells, we infected cultures of airway epithelial cells differentiated at air-liquid interface (ALI) with *M. catarrhalis* strain MC14 and monitored bacterial abundance and cellular responses. Over a 5-day incubation period, the number of cell-associated bacteria decreased by ~3 log units (**Figure 1A**). We did not observe any adverse effects of *M. catarrhalis* on bronchial epithelial cell morphology or ciliary motion throughout infection. Trans-epithelial resistance (TEER), a measure of epithelial barrier function, did not change at 24 h and tended to increase by 48 h post-infection (**Figure 1B**). This is in contrast to the effect of another common respiratory tract pathogen, *S. aureus*, which caused a decrease in TEER indicating damage to the epithelial barrier.

### Rhinovirus effects on *M. catarrhalis* association with epithelial cells

**Figure 2:**
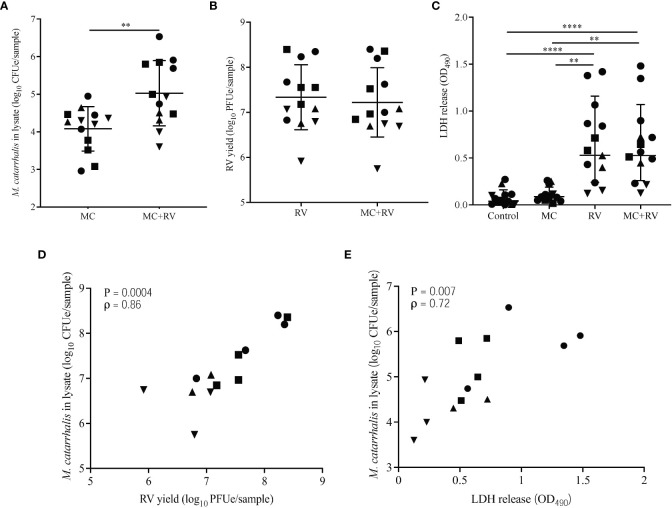
Rhinovirus increases *M. catarrhalis* survival and cell adhesion. ALI cultures from one donor were infected apically with RV-A16 for 2 hours followed by *M. catarrhalis* strain MC14. Cell-associated *M. catarrhalis* CFU counts from infected ALI cultures were determined every 24 h for 5 days. Data representative of 4 independent experiments. Symbols represent the geometric mean ± SD (panels **A**-**D**). Cultures from four different donors were infected with RV-A16 (2 hours), and then with *M. catarrhalis* for 48 hours. Cell lysates were analyzed for **(A)** *M. catarrhalis* genomic DNA abundance (CFUe), **(B)** RV RNA abundance (PFUe), and **(C)** cytotoxicity of epithelial cells (LDH release). Each symbol represents a separate cell line. Bars represent geometric mean ± SD (for CFUe and PFUe) or mean ± SEM (for LDH release). Results are from at least two independent experiments per donor (unpaired *t*-test or Mann-Whitney test; ^**^ *P*<0.01, ^****^ *P*<0.0001). Correlations were tested between cell-associated *M. catarrhalis* and **(D)** RV-A16 replication or **(E)** LDH release (Spearman’s correlation).

To determine the effects of RV infection on *M. catarrhalis* adherence and survival, we inoculated the apical surface of differentiated bronchial epithelium with RV-A16, followed 2 hours later by infection with a clinical isolate of *M. catarrhalis* (strain MC14). Repeat experiments in cells from four epithelial cell donors demonstrated that RV-A16 infection significantly increased cell-associated *M. catarrhalis* (geometric means for MC = 2.2×10^4^, MC+RV = 4.9×10^5^; *P* = 0.003) (**Figure 2A**). Notably, *M. catarrhalis* did not influence RV replication (**Figure 2B**). RV infection caused significant cytotoxicity, as measured by LDH release (mean for control = 0.08, MC = 0.11, RV = 0.67, MC+RV = 0.64; control vs RV or MC+RV, *P* < 0.0001) (**Figure 2C**) that was not altered by *M. catarrhalis.* Both RV replication (ρ = 0.86, *P* = 0.0004) (**Figure 2D**) and cytotoxicity (ρ = 0.72, *P* = 0.007) (**Figure 2E**) were positively correlated with cell-associated *M. catarrhalis.* These findings suggest that RV replication promotes *M. catarrhalis* attachment or persistence. It is of note that of the four BEC donors that we analyzed, two of them (denoted by circle and squares), had greater bacterial burden with RV co-infection than the other donors. These donors also had more RV replication and RV-induced cytotoxicity. These observations suggest that some individuals may be more susceptible to *M. catarrhalis* colonization during RV infection.

### Cellular response to *M. catarrhalis*

**Figure 3:**
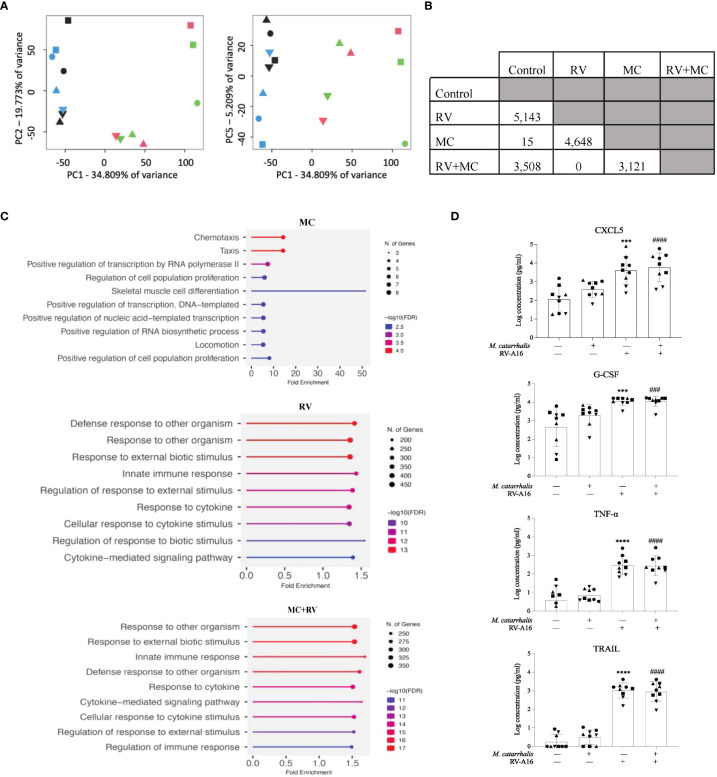
Gene expression and cytokine analyses of ALI cultures infected with RV and *M. catarrhalis.* ALI cultures from epithelial cell were infected with RV-A16, *M. catarrhalis* or both as in previous experiments. Principal component analysis of RNA-seq data indicates clustering of samples by treatment. **(A)** PC1 separates RV treated samples from non-RV treated samples while PC5 separates *M. catarrhalis* treated from *M. catarrhalis* untreated samples. The treatment applied to ALI cultures is represented by symbol color: control (blue), RV (green), MC (black), and MC+RV (red). The donors are coded by symbol shape. **(B)** Differentially expressed genes in ALI cultures from a single epithelial cell donor infected with RV, *M. catarrhalis* (MC) or both (RV+MC). RNA-seq analysis, conducted using linear regression modeling, indicated differentially expressed genes between each group except between the RV treated and the RV+*M. catarrhalis* treated samples. For each comparison, the number indicates the number of differentially expressed genes after multiple testing correction per analysis (FDR < 0.05). **(C)** Gene enrichment analysis of differentially expressed genes following infection with RV, *M. catarrhalis* (MC) or both (RV+MC) using ShinyGO 0.76 (Ge et al., 2020). The pathways were sorted by -log10 (FDR) values with aunpn FDR cutoff of 0.05. The dotplots show the top 10 enriched pathways for each treatment. **(D)** Cytokine levels were measured in the medium of ALI cultures infected with RV-A16 and *M. catarrhalis*. Bars represent geometric mean + SD. ^#^Control vs *M. catarrhalis* + RV; ^*^Control vs RV; (n = minimum of two per donor; ordinary one-way ANOVA; three symbols P < 0.001, four symbols P < 0.0001).

We next tested whether RV and/or *M. catarrhalis* infection influenced epithelial gene expression. Using principal component analysis (PCA), PC1 was related to RV infection and PC5 was related to *M. catarrhalis* infection (**Figure 3A**). RV induced a robust transcriptional response (5143 mRNAs), while *M. catarrhalis* alone upregulated only 15 mRNAs. There were no significant differences in gene expression between cells infected with RV vs. RV+MC (**Figure 3B**). Gene Ontology (GO) enrichment analysis of significantly expressed genes revealed that RV induced a number of innate immune and antiviral pathways. **Figure 3C** shows the top 10 enriched pathways for each treatment. *M. catarrhalis* downregulated the expression of 15 genes that are associated with chemotaxis and cell proliferation. Among them were the transcription factors early growth response (*EGR*) 1, 2, and 3, which are involved in mitogenesis and fibrinogenesis suggesting that *M. catarrhalis* could inhibit epithelial regeneration.

We next tested whether *M. catarrhalis* altered the epithelial immune responses to RV. RV-A16 infection alone significantly increased secretion of chemokines (CXCL5, CXCL10, eotaxin-3, and G-CSF) and proinflammatory cytokines (IL-1β, IL-6, and TNF-α). RV-A16 infection also led to the secretion of TNF-related apoptosis-inducing ligand (TRAIL) which activates cell death by binding to the TRAIL receptor (Girkin et al., 2017). **Figure 3D** is a representation of the cytokines that were measured. As expected, RV-A16 infection also led to the secretion of the anti-viral cytokine IL-28A (IFN-λ2). *Moraxella catarrhalis* alone did not significantly induce cytokine secretion from the epithelium and did not alter RV-induced cytokine secretion.

### Effects of RV species and bacterial isolate on cell-associated *M. catarrhalis*

**Figure 4:**
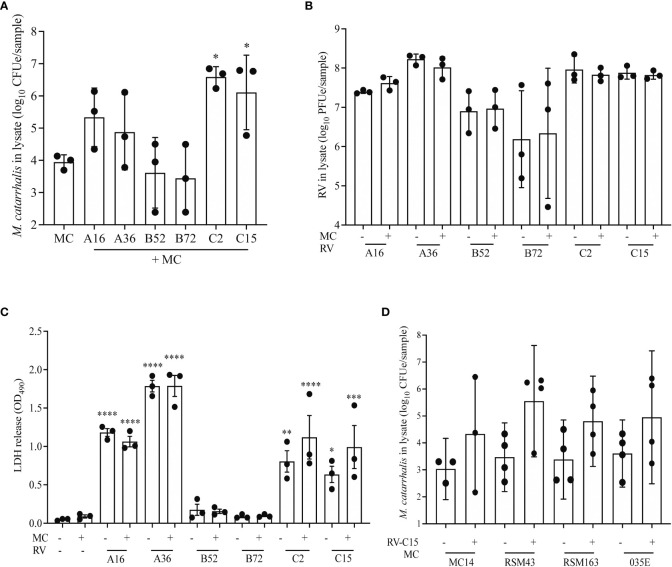
RV-A and RV-C, but not RV-B, cause increased adherence of *M. catarrhalis.* Differentiated cell cultures from two epithelial cell donors were infected with two representative types from each RV species (A16, A35, B52, B72, C2 and C15) **(A–C)** followed by the addition of *M. catarrhalis* strain MC14. After co-infection the following parameters were measured: **(A)** Adherent *M. catarrhalis* in cell lysate (*P* vs. uninfected control [“MC”]), **(B)** RV replication (*P* value vs B72), and **(C)** cytotoxicity (*P* vs. uninfected control [“MC-/RV-”]). Bars represent geometric mean ± SD. Results are from 3 independent experiments (ordinary one-way ANOVA; ^*^ *P* < 0.05, ^**^ *P*<0.01, ***P<0.001, ^****^ *P*<0.0001). **(D)** Following infection with RV-C15, four strains of *M. catarrhalis* (MC14, RSM43, RSM163, and 035E) were added to the culture. After co-infection, quantitative PCR was used to quantify the cell-associated *M. catarrhalis*. Bars represent geometric means ± SD (n = minimum of 3 per strain; unpaired t-test).

RV-A and RV-C cause more severe respiratory illness and are more often associated with increased detection of *M. catarrhalis in vivo* compared to RV-B (Bashir et al., 2018). To evaluate the differential effect of RV species on replication and *M. catarrhalis* cell adhesion and cell association, we infected fully differentiated ALI cultures from a single bronchial epithelial cell donor with two types of each RV species (A16, A35, B52, B72, C2, and C15). Compared to RV uninfected cells, both RV-C types significantly increased cell-associated *M. catarrhalis* while RV-B types did not (*P* = 0.0003 vs RV-C2; *P* = 0.03 vs RV-C15) (**Figure 4A**). *M. catarrhalis* did not affect RV replication (**Figure 4B**). Compared to RV-B types, RV-A and C types also caused more cytotoxicity as indicated by the increase in LDH levels (**Figure 4C**; one-way ANOVA; A16 and A36 *P* < 0.0001, C2 *P* = 0.002, C15 *P* = 0.02 vs B72). To examine strain-specific effects on cell association, we next tested four different strains of *M. catarrhalis* (three clinical isolates and laboratory strain 035E). RV-C15 infection induced increased adhesion of all of the bacterial strains to AECs. None of the strains caused significant epithelial cell death or augmented RV-driven cell death (**Figure 4D**).

To identify the factors that contribute most to *M. catarrhalis* cell association, we used a mixed-effects model that included RV species, RV replication, epithelial cell donor, and cytotoxicity. In this model *M. catarrhalis* cell association was significantly related to RV species (C > A > B, *P* = 0.003) and RV replication (*P* = 0.05).

### *Moraxella catarrhalis* adheres to epithelial cells infected with RV

**Figure 5:**
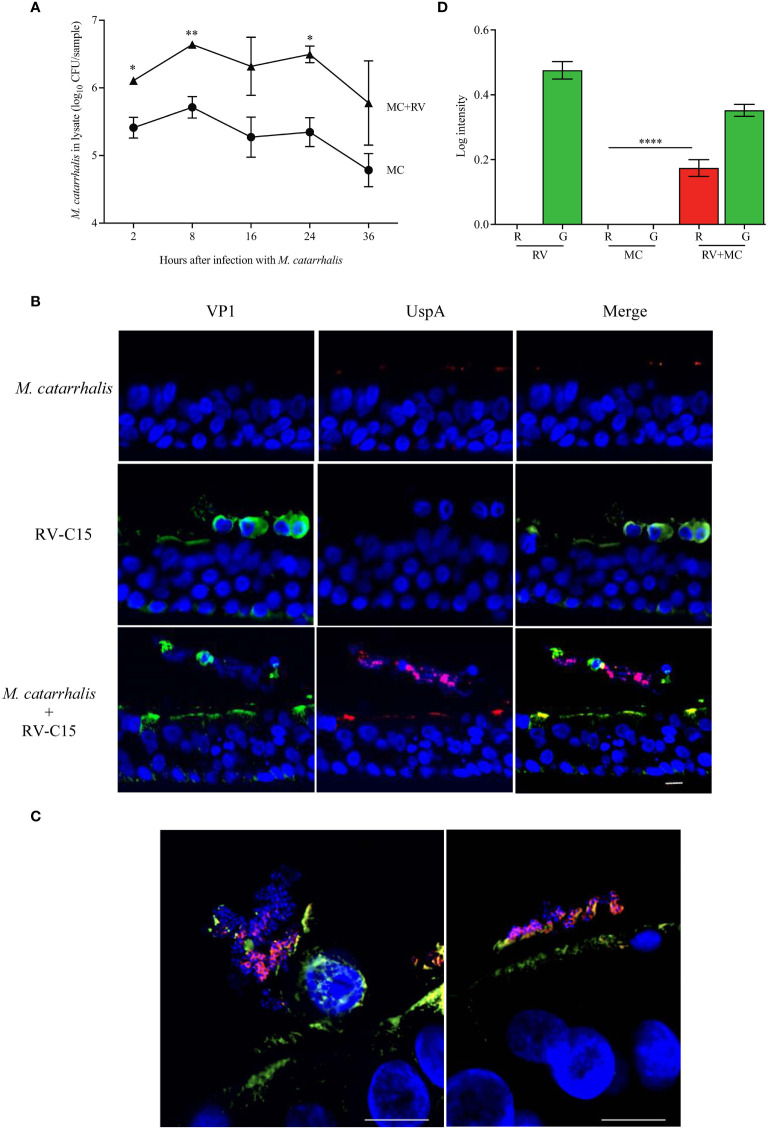
*M. catarrhalis* adheres to epithelial cells undergoing cell death due to RV-C15 infection. **(A)** ALI cultures were infected with RV-C15 (16 hours) and then with *M. catarrhalis* MC14. Live CFU counts were obtained by plating dilutions of apical washes taken after incubation with *M. catarrhalis* for 2, 8, 16, 24, and 36 hours. Symbols represent geometric mean ± SD (*n* = 4; multiple unpaired t-tests. For **(B, C)**, differentiated epithelial cell cultures were infected with RV-C15 (16 hours), *M. catarrhalis* MC14 (8 hours), or sequentially with RV-C15 (16 hours) followed by *M. catarrhalis* (8 hours). Representative histological sections were imaged (*M. catarrhalis*, red; RV-C15, green; DNA, blue) using an **(B)** 20✕ and a **(C)** 100✕ silicone immersion objective (n = 4; scale bar = 10 μm). **(D)** Intensity analysis of red (R = UspA) and green (G = VP1) signals from histological sections obtained in **(B)**. Bars represent geometric mean ± SD (n = 4, ****P < 0.0001).

As we were able to show that RV-C species had the greatest effect on *M. catarrhalis* cell association and epithelial outcomes such as cytotoxicity, we used RV-C15 as a representative to look at the timeline of infection. To determine how soon *M. catarrhalis* binds to RV-infected cells, we infected ALI cultures with RV-C15 for 16 h (approximately one replication cycle) and then added *M. catarrhalis* for 2-36 h. *Moraxella catarrhalis* had increased cell adhesion starting 2 h post-infection and persisting for at least 36 h post-infection (**Figure 5A**). As *M. catarrhalis* showed maximal adhesion at 8 h after infection, we used this time point to evaluate conditions affecting bacterial adhesion in subsequent experiments.

We next used immunohistochemistry to visualize *M. catarrhalis*-cellular interactions. Cells were infected with RV-C15 for 16 h and then incubated with *M. catarrhalis* for 8 h. Without RV infection, few bacteria adhered to the epithelium. In contrast, *M. catarrhalis* attached to the apical surface of RV-infected cells, particularly cells that stained positive for RV and that were extruded from the infected epithelium and are dying (**Figures 5B, C**). While *M. catarrhalis* has been reported to internalize into lung epithelial cell lines and primary cells by macropinocytosis (Slevogt et al., 2007), we did not observe internalized bacteria in RV-infected or uninfected cells. Analysis of the immunofluorescent signal intensities showed a significantly higher signal for UspA in the presence of RV-C15 co-infection (*P* < 0.0001) (**Figure 5D**).

### UspA is involved in the adhesion of *M. catarrhalis* to the respiratory epithelium

**Figure 6:**
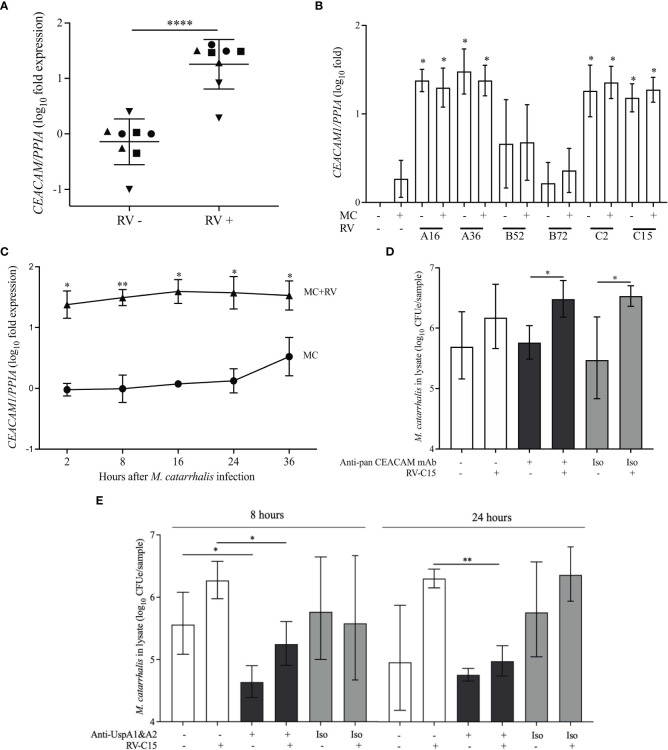
UspA1 and UspA2 contribute to the adhesion of M. catarrhalis to BECs To evaluate RV-induced *CEACAM1* expression in AECs, differentiated ALI cultures **(A)** from four epithelial cell donors were infected with RV-A16 for 2 hours and then with *M. catarrhalis* MC14 for 48 hours. Cell donors are represented by different symbols, and expression is relative to donor 1 (circles) (n = 2 per donor; unpaired t-test test). **(B)** ALI cultures from donor 1 were infected with RV species (A16, A35, B52, B72, C2 and C15) for 2 hours and then with *M. catarrhalis* MC14 for 48 hours. *CEACAM1* expression was evaluated by qPCR. For **(C)** ALI cultures were infected with RV-C15 for 16 hours and then with *M. catarrhalis*. *CEACAM1* expression was evaluated at 2, 8, 16, 24, and 36 hours following *M. catarrhalis* infection (*n* = 4, multiple unpaired t-tests). **(D)** Following RV-C15 infection for 16 hours, ALI cultures were incubated with mouse anti-pan *CEACAM* antibodies for 1 hour before adding *M. catarrhalis* MC14 for 8 hours. Cell-associated *M. catarrhalis* were quantified by qPCR. Bars represent geometric mean ± SD (*n* = 4, unpaired t-test). **(E)** *M. catarrhalis* strain MC14 was incubated with mouse anti-UspA antibodies for 1 hour and the mixture was added onto the apical surface of the epithelium for 8 or 24 hours following RV-C15 infection. Cell-associated *M. catarrhalis* was quantified by qPCR. White bars = no antibodies, black bars = anti-UspA antibodies, grey bars = isotype control (Iso). Bars represent geometric mean ± geometric SD (n = 4; unpaired t-test). *P < 0.05, **P<0.01, ****P<0.0001.

*M. catarrhalis* contains several OMPs that are important for adhesion. These include lipooligosaccharide (Spaniol et al., 2008), UspA1 (Lafontaine et al., 2000; Spaniol et al., 2008), UspA2 (Lafontaine et al., 2000), and Hag/MID (Bullard et al., 2005). Human airway epithelial cells express CEACAM1, CEACAM5, and CEACAM6 (Klaile et al., 2013) and UspA1 has been shown to bind to the N-terminus of CEACAM1 (Hill and Virji, 2003). *M. catarrhalis* adhesion can be prevented by using monoclonal antibodies that bind to the N-terminus of CEACAM1 (Hill and Virji, 2003; Green et al., 2011). RV-A16 infection significantly increased the expression of CEACAM in the epithelial cell donors with increased *M. catarrhalis* cell association (**Figure 5A**). Infection with RV-A and RV-C induced the expression of *CEACAM1* by ~30-fold, while RV-B and *M. catarrhalis* did not have an effect on *CEACAM1* expression (**Figure 5B**). Blocking CEACAM with mouse monoclonal antibodies D14HD11 that bind to the N-terminus of all CEACAMs, that have previously been used to inhibit *Neisseria meningitidis* adhesion (Green et al., 2011), did not inhibit *M. catarrhalis* cell adhesion (**Figure 6D**) despite being significantly upregulated beginning at 18 hours post-infection with RV-C15 (**Figure 5C**). We also used rat monoclonal antibodies YTH71.3 to CEACAM that have previously been used to inhibit *Moraxella catarrhalis* (Hill and Virji, 2003), but did not see any effect (data not shown). *Moraxella catarrhalis* did not influence CEACAM1 expression (**Figures 5B, C**). This is in contrast to a previous study that showed induction of CEACAM1 by *M. catarrhalis* (Klaile et al., 2013). In addition, fluorescent microscopy demonstrated that bacterial adhesion did not colocalize with CEACAM staining of RV-infected epithelial cells (Pearson’s correlation coefficient range = -0.09 to 0.14; *P* value range = 0.1 to 0.8) (**Figures S1A, B**). In contrast, blocking UspA1 and A2 with mouse monoclonal antibodies significantly reduced RV-induced *M. catarrhalis* adherence to airway epithelium at 24 h (**Figure 6E**). Interestingly, UspA1 and UspA2 showed minimal induction even at 36 h post-infection with RV-C15 infection (**Figures S2A, B**), showing that the increased adhesion of *M. catarrhalis* was not due to the increased expression of the outer membrane adhesion protein UspA.

## Discussion

Using a fully differentiated *in vitro* model of the human airway epithelium, we demonstrated that *M. catarrhalis*, an opportunistic pathogen of the human respiratory tract, was well tolerated and did not cause epithelial damage (**Figure 1**). During co-infection with RV-A or RV-C, there was a significant increase in cell-associated *M. catarrhalis* (**Figures 2**, **4**). This suggests that the increased replication rates for RV-A and RV-C compared to RV-B species leads to more epithelial damage which supports the cell association of *M. catarrhalis (* Nakagome et al., 2014). These findings support clinical observations in which infections with RV-A or RV-C, which cause more severe illness than RV-B, were associated with increased detection in nasal secretions of respiratory pathogens like *M. catarrhalis*, *H. influenzae*, and *S. pneumoniae (* Bashir et al., 2018). The epithelial cytokine and gene expression responses were driven by RV with little contribution from *M. catarrhalis* (**Figure 3**). We also found that *M. catarrhalis* preferentially associates with RV-infected cells that may be undergoing cell death (**Figure 5B, C**) and that the outer membrane adhesion protein UspA is involved in the attachment of *M. catarrhalis* to respiratory epithelial cells (**Figure 5**). Analysis of the immunofluorescent signal intensities showed a significantly higher signal for UspA in the presence of RV-C15 co-infection (*P* < 0.0001) (**Figure 5D**).

Our observations are consistent with other studies showing that viral infections can induce the adhesion of bacterial pathogens. Respiratory syncytial virus infection increases the virulence of *S. pneumoniae* by directly binding to bacteria and upregulating genes required for virus binding and bacterial invasiveness (Smith et al., 2014). Influenza virus also directly interacts with *H. influenzae*, *M. catarrhalis*, *S. aureus*, and *S. pneumoniae*, increases adhesion to respiratory epithelial cells, and induces middle ear translocation of bacteria to promote otitis media (Rowe et al., 2019). *Moraxella catarrhalis* produces several adhesins, including UspA1, UspA2, UspA2H, and Hag, and these molecules mediate binding to airway tissues. UspA1 and UspA2 are relatively conserved and can be detected in most nasopharyngeal isolates of *M. catarrhalis (* Meier et al., 2002). UspA1 binds to extracellular matrix proteins laminin (Tan et al., 2006) and fibronectin (Tan et al., 2005) as well as to CEACAM1, which is a transmembrane protein expressed by airway epithelial cells (Hill and Virji, 2003; Conners et al., 2008). UspA2, also binds to extracellular matrix proteins such as collagen (Singh et al., 2016), laminin (Tan et al., 2006), fibronectin (Tan et al., 2005), and vitronectin (Singh et al., 2010), while the hybrid UspA2H has mixed binding properties. Hag/MID can bind to collagen (Balder et al., 2009). In our studies, RV-A and RV-C infection induced CEACAM1 but blocking CEACAM did not inhibit *M. catarrhalis* binding to airway epithelial cells (**Figures 6B, D**). The respiratory epithelium expresses CEACAM1, 5, and, 6 (Klaile et al., 2013). Soluble CEACAM5 and CEACAM6 can be detected in normal bronchial mucus (Matsuoka et al., 1990; Klaile et al., 2013), and secreted isoforms could inhibit adhesion to CEACAM expressed on the surface of the respiratory epithelium. As RV can increase the synthesis of fibronectin (Wang et al., 2009) and damage the epithelial barrier (Looi et al., 2018) allowing access to fibronectin and other matrix proteins (Singh et al., 2016), it is also possible that *M. catarrhalis* binds to these proteins which become more abundant following epithelial damage.

We observed that *M. catarrhalis* formed clusters around dying cells on the apical surface of the epithelium and in cell debris in the mucin layer (**Figure 6**). Therefore, it is possible that cellular damage also leads to the release of nutrients providing an environment conducive for *M. catarrhalis* growth as seen in the GI tract, where colonocytes undergoing apoptosis release small molecules that are a source of nutrition for bacterial growth, a process known as death-induced nutrient release (Anderson et al., 2021).

Upper airway colonization with *M. catarrhalis* is widespread in healthy preschool children (Verhaegh et al., 2011), and *M. catarrhalis* increases in abundance during RV infections and asthma exacerbations (Kloepfer et al., 2014; Bashir et al., 2018). Bacterial invasion of host epithelia is a strategy used by bacteria for persistence and dissemination leading to more severe and recurrent disease (Alexander and Hudson, 2001; Asmat et al., 2014; Murphy et al., 2004). UspA1 and lipooligosaccharide (LOS) also contribute to cellular invasion (Spaniol et al., 2008). Our observations indicate that *M. catarrhalis*, by itself, does not invade or damage the respiratory epithelium or induce epithelial cytokine responses. In contrast, RV infection caused the secretion of cytokines, including chemoattractants for neutrophils (CXCL5 and CXCL10) and eosinophils (CCL26 [eotaxin-3]) and the tumor necrosis factor-related apoptosis-inducing ligand (TRAIL), which has been linked in mouse models to airway hyperactivity, cellular infiltration and anti-viral IFN responses (Girkin et al., 2017). Co-infection with RV and *M. catarrhalis* did not cause a synergistic cytokine response, suggesting that *M. catarrhalis* induces minimal responses from airway epithelial cells, with or without RV infection. Nevertheless, RV infection induces neutrophil chemotactic factors and the influx and activation of neutrophils. Strong inflammatory and neutrophilic responses have been observed in a mouse model of airway *M. catarrhalis* infection with more intense responses when infection occurred in house dust mite sensitized animals (Alnahas et al., 2017). In a cohort of asymptomatic neonates, whose upper airways were colonized with *M. catarrhalis* or other pathogens, cytokine analysis of the nasal mucosal lining fluid revealed a mixed Th1/Th2/Th17-type response with increased levels of IL-8 and IL-17, which cause neutrophil recruitment and activation (Følsgaard et al., 2013). This suggests that neutrophilic inflammation, induced by RV and amplified by *M. catarrhalis*, could worsen the RV-induced epithelial damage and increase illness severity. In our experiments, RV-C caused significantly more cell association than other RV species, which corresponds with clinical observations (Bashir et al., 2018). This raises the possibility that RV-C induces a unique environment that is preferred by *M. catarrhalis.*

Our study’s main strength was the co-infection model using fully differentiated respiratory epithelium, providing a multicellular model of the cellular responses to bacterial and viral infection. We also tested multiple types of RV and different strains of *M. catarrhalis* to understand their similarities and differences. The RV were all cloned from clinical isolates, and three of the four *M. catarrhalis* strains used in the study were isolated from clinical samples to mimic conditions *in vivo*. Our study also has limitations that should be considered in interpreting these results. The small number of cell donors in our study limited the power to explore individual variability. Second, multicellular models containing epithelial and immune cells are needed to test the effects of *M. catarrhalis* adhesion on leukocyte-mediated inflammation. Although we did not observe an epithelial cytokine response to *M. catarrhalis* infection, RV infection recruits neutrophils and other leukocytes into the airway. It is possible that interactions between epithelial cells, adherent bacteria and neutrophils could amplify inflammatory responses at the epithelial surface to increase the severity of illness. In this study we only looked at a possible interaction between CEACAM and UspA proteins. It is possible that use of polyclonal blocking antibodies instead of monoclonal antibodies as used in this study, may give better blockage of the bacterial binding epitopes of CEACAM. We have not looked at possible interactions between other OMPs and extracellular matrix proteins and therefore cannot exclude the possibility that these proteins may play a role in RV-induced cell association of *M. catarrhalis*.

In conclusion, our study revealed that *M. catarrhalis* increases association with respiratory epithelial cells during RV infection by adhering to infected cells and those extruded from the epithelial surface. These findings suggest that virus-induced cell death promotes adherence and survival of this common bacterial pathogen. The lack of *M. catarrhalis*-induced inflammation or cytotoxicity further suggests that this bacterium could intensify the severity of viral illness by focusing leukocyte-mediated inflammation at the epithelial surface. This suggests a possible mechanism by which RV and *M. catarrhalis* co-infection increases the severity of respiratory illness in children.

## Methods

### Bacteria and viruses

*Moraxella catarrhalis* strain MC14 was isolated from a clinical specimen by the University of Wisconsin Clinical Pathology Laboratory. In addition, *M. catarrhalis* strains RSM43 and RSM163 were isolated from the nasal secretions of children with acute respiratory illnesses (Kloepfer et al., 2014), and strain 035E was originally isolated from the middle ear fluid of a patient with otitis media (Unhanand et al., 1992). For epithelial inoculation, we cultured *M. catarrhalis* from a frozen glycerol stock onto chocolate agar plates that were incubated at 37 °C in a 5% CO_2_ environment for 48 h. Bacteria were re-streaked onto fresh chocolate agar plates and cultured for a further 24 h.

RV strains were cloned from clinical isolates and grown as described previously (Nakagome et al., 2014). The types used in the study were RV-A16, RV-A36, RV-B52, RV-B72, RV-C2, and RV-C15 (Bochkov et al., 2011; Lee et al., 2012; Nakagome et al., 2014).

### Isolation of genomic DNA from *M. catarrhalis* isolates

*Moraxella catarrhalis* was cultured overnight in 3 ml brain-heart-infusion (BHI) broth at 37 °C. The cultures were centrifuged at 21,130 × g for 5 min at ambient temperature and washed in 1 ml of Buffer 1 (150 mM NaCl, 10 mM EDTA, 20 mM Tris-HCl, pH 8). The cells were resuspended in 1 mL of fresh Buffer 1 with 10 µL of 100 mg/mL RNase A and 163 µl of 10% [wt/vol] SDS. The samples were incubated at 37 °C for 90 min. Subsequently, 20 µl of 20 mg/ml proteinase K were added and the samples were incubated at 65 °C for 20 min. After an additional incubation at 37 °C for 16 h to ensure complete lysis, genomic DNA was purified using standard phenol-chloroform extraction and precipitated with isopropanol. Genomic libraries for Illumina MiSeq 2×150-bp paired-end sequencing were prepared and sequenced by the University of Wisconsin-Madison Biotechnology Center. The raw reads were corrected using fastp 0.20.0 (Chen et al., 2018) and draft genomes were generated using SPAdes v3.11.0 with default parameters (Prjibelski et al., 2020).

### Culture of airway epithelium at air-liquid interface

Human bronchial and tracheal epithelial cells were obtained from residual tissue from lungs destined for transplantation in collaboration with the University of Wisconsin - Health Lung Transplant Program. The protocol was reviewed by the University of Wisconsin Institutional Review Board and was deemed “not human subjects research.” Cryopreserved aliquots of cells were thawed and expanded as monolayers in PneumaCult-Ex Plus Medium (StemCell Technologies) supplemented with 0.1% [vol/vol] gentamicin (Sigma) and 0.1% [vol/vol] fluconazole (Novaplus). Once the cells reached 80% confluence, they were transferred to 12-well plates with Transwell semi-permeable inserts (Corning 3460) and were allowed to differentiate in PneumaCult-ALI medium (StemCell Technologies) supplemented with 0.1% [vol/vol] gentamicin (Sigma) and 0.1% [vol/vol] fluconazole (Novaplus) at the air-liquid interface for at least 21 days when ciliary motion was observed. The culture medium was changed to an antibiotic-free medium containing 0.05% [vol/vol] hydrocortisone 48 hours prior to RV infection. All ALI cultures were used between 28 – 35 days of air lifting.

### RV and *M. catarrhalis* infection of epithelial cultures

The apical surface of the epithelium was washed with pre-warmed phosphate-buffered saline (PBS) supplemented with 100 mg/l of Ca^2+^ and Mg^2+^and then infected with 10^7^ PFU of RV (in 50 μl of antibiotic-free culture medium) at a multiplicity of infection (MOI) of 10. The cells were incubated at 34 °C for 2 h, after which the apical surface was washed (3×) with pre-warmed PBS (containing Ca^2+^ and Mg^2+^). For infection of differentiated cultures, 3-4 colonies of *M. catarrhalis* were picked from the growth plates and used to make a bacterial suspension in pre-warmed PBS at a concentration of 2 McFarland units (~6×10^8^ CFU/ml). The apical surface was then infected with 50 μl of the *M. catarrhalis* suspension (~3×10^7^ CFU) and incubated at 37 °C for 48 h.

For time-course experiments in **Figure 1A**, differentiated respiratory epithelium was infected with 10^7^ PFU of RV-A16 at 34 °C for 2 h and then with 3×10^7^ CFU of *M. catarrhalis.* At specified timepoints after *M. catarrhalis* infection, the apical surface was washed with 0.5 ml of pre-warmed PBS (with Ca^2+^ and Mg^2+^) and cell lysates were collected in 350 µl RLT Plus Buffer from AllPrep DNA/RNA Mini Kit (Qiagen) containing 0.5% [vol/vol] Reagent DX (Qiagen) for quantitative PCR. To quantify cell associated live bacteria in **Figure 5A**, ALI cultures were infected with 10^7^ PFU of RV-C15 at 34 °C for 2 h and then for at 37 °C for a further 16 h before infecting with 3×10^7^ CFU of *M. catarrhalis* for specified durations. The apical surface washed with 0.5 ml of pre-warmed PBS (with Ca^2+^ and Mg^2+^). Cell associated bacteria were quantified by adding 100 μl of 1% [vol/vol] saponin on to the apical epithelial surface at RT for 15 min, washing with 0.5 ml of warmed PBS (with Ca^2+^ and Mg^2+^) and plating the serially diluted washes on chocolate agar. The colony counts were done 48 h after plating.

For trans-epithelial electrical resistance (TEER) measurements, the apical surface of ALI cultures were infected with 50 μl of the *M. catarrhalis* suspension (~3×10^7^ CFU) that was prepared as described earlier and incubated at 37 °C for 24 or 48 h. As a control, a clinical isolate of *Staphylococcus aureus* was cultured from a frozen glycerol stock onto blood agar plates that were incubated at 37 °C in a 5% CO_2_ environment for 48 h. Bacteria were re-streaked onto fresh blood agar plates and cultured for a further 24 h. For infection of ALI cultures, a colony of *S. aureus* was picked from the growth plates and used to make a bacterial suspension in pre-warmed PBS at a concentration of 2 McFarland units (~6×10^8^ CFU/ml). The apical surface of the ALI cultures were then infected with 50 μl of the suspension (~3×10^7^ CFU) and incubated at 37 °C for 24 or 48 h.

### Gentamycin protection assay for quantification of intracellular *M. catarrhalis*

The apical surface of the epithelium was washed with pre-warmed phosphate-buffered saline (PBS) supplemented with 100 mg/l of Ca^2+^ and Mg^2+^and then infected with 10^7^ PFU of RV (in 50 μl of antibiotic-free culture medium) at an MOI of 10. The cells were incubated at 34 °C for 2 h, after which the apical surface was washed (3×) with pre-warmed PBS (containing Ca^2+^ and Mg^2+^). For infection of differentiated cultures, 3-4 colonies of *M. catarrhalis* were picked from the growth plates and used to make a bacterial suspension in pre-warmed PBS at a concentration of 2 McFarland units (~6×10^8^ CFU/ml). The suspension was diluted 100-fold to obtain a suspension with a concentration of 6×10^6^ CFU/ml. The apical surface was then infected with 50 μl of the 6×10^6^ CFU/ml *M. catarrhalis* suspension (~3×10^5^ CFU) and incubated at 37 °C for 48 h. Following the incubation period, the apical surface was washed with 0.5 ml of pre-warmed PBS and 100 µl of 100 µg/ml of gentamicin was added to the apical surface and incubated at 37 °C for 1 hour. The apical surface was washed with 0.5 ml of pre-warmed PBS and 100 µl of 1% saponin was added to the apical surface for 15 minutes at room temperature. The apical surface was washed again with 0.5 ml of pre-warmed PBS and serial dilutions of the washes were plated to determine live intracellular CFU counts.

### TEER measurement

Culture medium in outer wells of the Transwell plate were removed and replaced with 1 ml of warmed DMEM/F12 medium (Gibco) and 0.5 ml of DMEM/F12 in the insert (on apical surface of cells). The cells and medium were allowed to incubate at RT for 15 min. The TEER was measured using an epithelial Voltohmmeter (EVOM2; World Precision Instruments, USA).

### Quantitative PCR

Nucleic acids from the epithelium (± bacteria) were harvested from the inserts using 350 µl RLT Plus Buffer from AllPrep DNA/RNA Mini Kit (Qiagen) containing 0.5% [vol/vol] Reagent DX (Qiagen). The lysate was transferred to PowerBead tubes (Qiagen) for bead beating (5 min, 50 oscillations/s, Qiagen TissueLyser LT). RNA and DNA were extracted as separate fractions (AllPrep^®^ DNA/RNA Mini Kit). *Moraxella catarrhalis copB* and RV RNA levels were quantified by real-time PCR using specific primers (**Table 1**) as previously described (Greiner et al., 2003; Bochkov et al., 2011). The CFUe measurements were calibrated by using a standard curve. To generate the standard curve, *M. catarrhalis* strain MC14 was cultured overnight in tryptic soy broth. The suspension was serially diluted and plated to obtain CFU counts. Two milliliters of the original suspension were centrifuged at 13,000 rpm for 30 minutes. The resulting bacterial pellet was lysed by adding warmed (55 °C) Solution PM1 followed by DNA extraction (AllPrep^®^ Power Viral Kit, Qiagen). The extracted DNA was serially diluted to make a standard curve corresponding to 10^6^ to 10^2^ CFU.

**Table 1:** Primers and probes used in the study.

Name	Sequence 5’ ➔ 3’	Description
RV1A16	CCTCCGGCCCCTGAAT	RV
R848	AAACACGGACACCCAAAGTAGT	RV
*UspA1-F*	AGGGATCCAACGACGGTCCAAGA TGG	UspA1
*UspA1-R*	AGGGATCCCCTGCCACCTAAAGCCTTG	UspA1
*UspA2-F*	CGGGATCCCTTCTCCCC CTAAAAATCGCTG	UspA2
*UspA2-R*	AGGGATCCCGCTGTATGCCGCTAC TCGCAGCT	UspA2
*copB-F*	GTGAGTGCCGCTTTACAACC	copB
*copB-R*	TGTATCGCCTGCCAAGACAA	copB
*copB* probe	NED-TGCTTTTGCAGCTGTTAGCCAGCCTAA-MGB-NFQ	copB
*CEACAM1-F*	TGCTCTGATAGCAGTAGCCCT	CEACAM1
*CEACAM1-R*	TGCCGGTCTTCCCGAAATG	CEACAM1
*PPIA-F*	CCCACCGTGTTCTTCGACATT	PPIA
*PPIA-R*	GGACCCGTATGCTTTAGGATGA	PPIA

For the quantification of mRNA expression, cDNA was prepared from total RNA extracted from ALI culture lysates using TaqMan™ Reverse Transcription Reagents (Applied Biosystems). *CEACAM1* mRNA expression was measured using specific primers (PrimerBank ID 329112546c1). *UspA1* and *UspA2* expression were quantified using specific primers as described previously (Lafontaine et al., 2001) (Wang and Seed, 2003). Gene expression was quantified using Power SYBR Green PCR Master Mix (Applied Biosystems) on an Applied Biosystems™ 7500 Real-time PCR system and normalized against the expression of the stable housekeeping gene *PPIA* (PrimerBank ID 114520617c1). The expression was normalized against *copB*. All primers used in the study are given in **Table 1**.

### Gene expression analysis

RNA was converted to cDNA using the SMART-Seq v4 Ultra Low Input RNA kit for sequencing (Takara Bio cat. 634898). Standard library preparation was completed using the Illumina Nextera XT DNA Library Preparation Kit and library quality and concentration were assessed using an Agilent 2100 bioanalyzer. Indexed samples were pooled and sequenced on the Illumina Hi-Seq 4000 with 100 base-pair, paired-end sequencing to a minimum of 10 million mapped reads per sample. RNA-seq reads were mapped to genome assembly GRCh37(hg19) reference sequence using STAR (version 2.6.1) (Dobin et al., 2013). Read counts were adjusted to counts per million and normalized using TMM normalization (Robinson and Oshlack, 2010). PCA was conducted in R (version 3.6.2) using prcomp. voom was used to adjust for significant technical covariates (Law et al., 2014). Genes significantly associated with RV or *M. catarrhalis* treatment were identified using Limma linear mixed modeling in R (Limma R package version 3.5 https://bioconductor.org/packages/release/bioc/html/limma.html). An FDR-adjusted p value of 0.05 was used as a significance threshold, using the method of Benjamini and Hochberg (Hochberg and Benjamini, 1990).

### Cytotoxicity measurement

Cellular cytotoxicity was estimated by measuring extracellular lactate dehydrogenase using a CytoTox 96^®^ Non-Radioactive Cytotoxicity Assay, (Promega) following manufacturer’s instructions.

### Cellular responses to infection

The cytokine levels in the culture medium in the basal compartment of the ALI culture system were measured by multiplex ELISA using a MILLIPLEX MAP 10-plex Human Cytokine/TH17 Mag Kit (Millipore Sigma). Epithelial cell gene expression was assessed by using 2×150 bp paired-end NovaSeq platform at the University of Wisconsin – Madison Biotechnology Center’s Gene Expression Center Core Facility (Madison, WI) (Research Resource Identifier – RRID : SCR_017757) for RNA library preparation and the DNA Sequencing Facility (RRID : SCR_017759) for sequencing.

Gene enrichment analysis of differentially expressed genes was done using the ShinyGO 0.76 online software (Ge et al., 2020). The pathways were sorted by -log10 (FDR) values with an FDR cutoff of 0.05.

### Blocking cellular or bacterial surface proteins

To block UspA1 and A2, we incubated 50 μl of a 2 McFarland unit *M. catarrhalis* suspension (~3×10^7^ CFU) with an equal volume of undiluted culture supernatant from the mouse hybridoma 17C7 (ATCC HB-11093) (Helminen et al., 1994; Aebi et al., 1997), (37 °C, 1 h). The mixture was then added to the apical surface of differentiated airway epithelial cells, with or without RV infection. *Moraxella catarrhalis* was quantified by qPCR at 8 and 24 h post-infection.

To block CEACAM, airway epithelial cells were infected with RV-C15 for 16 h. After washing with 0.5 ml of pre-warmed PBS, mouse anti-pan CEACAM antibody D14HD11 (ab4567, Abcam; 100 μl of 100 ng/ml) or of rat monoclonal anti-CEACAM antibody YTH71.3 (Santa Cruz Biotechnology; 100ul of 100 ng/ml) was added to the apical surface and incubated at 37 °C for 2 h. We washed the cells three times and then incubated them with 50 μl of a 2 McFarland unit *M. catarrhalis* (~3×10^7^ CFU, 37 °C, 8 h or 24 h).

### Immunohistochemistry

Mature ALI cultures were fixed in 10% [vol/vol] neutral-buffered formalin, embedded in paraffin wax and sectioned. The sections were deparaffinized by heating (60 °C, 20 min) and then transferring to 3 changes of xylenes. The sections were rehydrated using graded ethanol solutions (100%, 95%, 80%, 70% and 50% [vol/vol]) and deionized water. Antigen retrieval was performed by heating at 80 °C in a water bath for 2.5 h. Sections were permeabilized (1% normal goat serum with 0.4% [vol/vol] Triton X-100, 5 min), blocked (PBS with 0.1%[vol/vol] Tween 20 and 5% [vol/vol] goat serum), and then incubated (overnight, 4 °C) with antibodies to RV-C15 VP1 (1:200) or mouse anti-pan CEACAM antibody D14HD11 (ab4567, Abcam) (1:2000) diluted in 1% [vol/vol] normal goat serum containing 0.1% [vol/vol] Tween 20. After washing (PBS with 0.1% [vol/vol] Tween 20, 5 min), sections were incubated with the secondary antibody (Alexa Fluor^®^ 488 goat anti-mouse), blocked for 1 h with 5% mouse serum in PBS, and incubated with the primary antibody to *M. catarrhalis* UspA (Helminen et al., 1994; Aebi et al., 1997)(undiluted culture supernatant from the mouse hybridoma 17C7 - ATCC HB-11093, overnight, 4 °C). After washing three times, the second secondary antibody (Alexa Fluor^®^ 555 goat anti-mouse) was added (1 h, ambient temperature). The sections were counterstained with 4’,diamidine-2’-phenylindole dihydrochloride at 1:1000 for 15 min before mounting with FluorSave Reagent (EMD Millipore). Images were acquired on a Nikon A1 R confocal microscope (20× objective) and on a Nikon AX R point scanning confocal system equipped with 4 high-sensitivity GaAsP detectors using a Plan Apo lambda S 100× silicone immersion objective (NA 1.35) at 2048 x 2048 pixel density and 4x scan zoom. The 100× images were processed with Denoise.ai after acquisition. Image analysis was done using RGB Profile Plot **(** **Figure 5D** **)** and Colocalization Finder **(Figure S1B)** plugins on ImageJ v1.53t.

### Statistical analysis

Statistical analyses were performed using GraphPad Prism v.9.3.1 (GraphPad Software, Inc). Student’s ordinary *t*-test or the Mann-Whitney test were used to compare two groups. When more than two groups were compared, ordinary one-way ANOVA was used for the analysis. Correlation coefficients were calculated using Spearman’s rho statistic. To identify factors that significantly predict *M. catarrhalis* cell association, linear regression modeling with random slope and random intercept using SAS procedure Mixed was conducted. The likelihood ratio test (LRT) was used to for model selection. A p-value < 0.05 was considered statistically significant. SAS software (v.9.4, SAS Institute, Cary, NC) was used to develop the mixed-effects model.

## Data availability statement

The datasets presented in this study can be found in online repositories. The name of the repository/repositories and accession number(s) can be found below: PRJNA875903.

## Author contributions

JG, RB-S and ED conceived the study. ED and RB-S planned and conducted experiments. RS provided clinical bacterial isolates and performed bacterial whole genome sequencing. YB provided virus preparations. BH and CO planned and performed epithelial gene expression studies and analysis. CK, TM, and CC provided guidance on study design, experimental methods and data interpretation. ED, RB-S, and ZZ performed statistical analysis. All authors contributed to the final draft of the manuscript and approved the final version. ED and RB-S contributed equally to this study.
