# Supplementary material for: Rhinovirus increases Moraxella catarrhalis adhesion to the respiratory epithelium
Source: Front Cell Infect Microbiol. 2023 Jan 17;12:1060748. doi: 10.3389/fcimb.2022.1060748 (PMC9886879; doi:10.3389/fcimb.2022.1060748)
Supplement: Supplementary file 1 [file DataSheet_1.pdf]

(A)

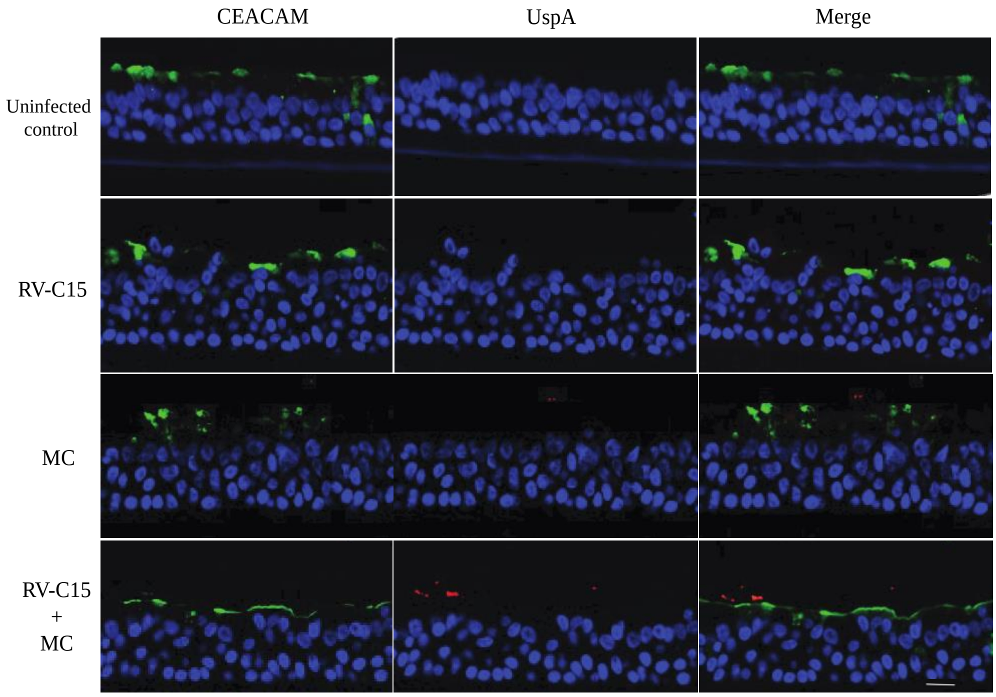

(B)

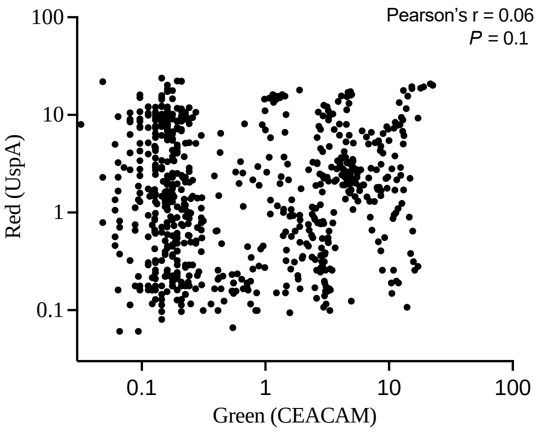

Fig. S1: (A) ALI cultures infected with RV-C15 for 2 hours, *M. catarrhalis* or both for 48 hours and were labeled with rabbit pan anti-CEACAM antibodies (green) and mouse anti-UspA (red) antibodies. DNA was stained with DAPI (blue). Representative histological sections were imaged using a 20× objective from at least 3 independent experiments. Scale bar = 20 µm. **(B)** Representative scatterplot showing distribution of CEACAM and UspA signals in the RV-C15+MC infected epithelium from **(A)** (n = 5; Pearson's correlation range = -0.09 to 0.14; *P* value range = 0.1 to 0.8).

(A)

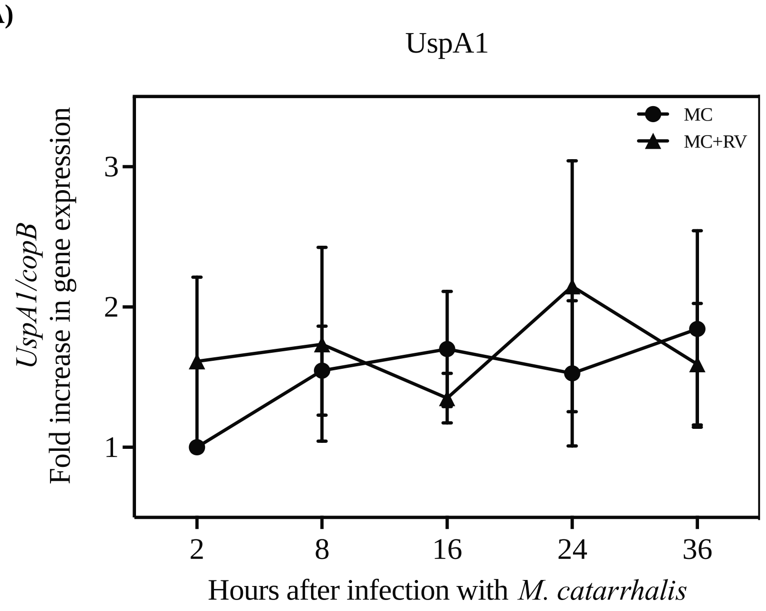

(B)

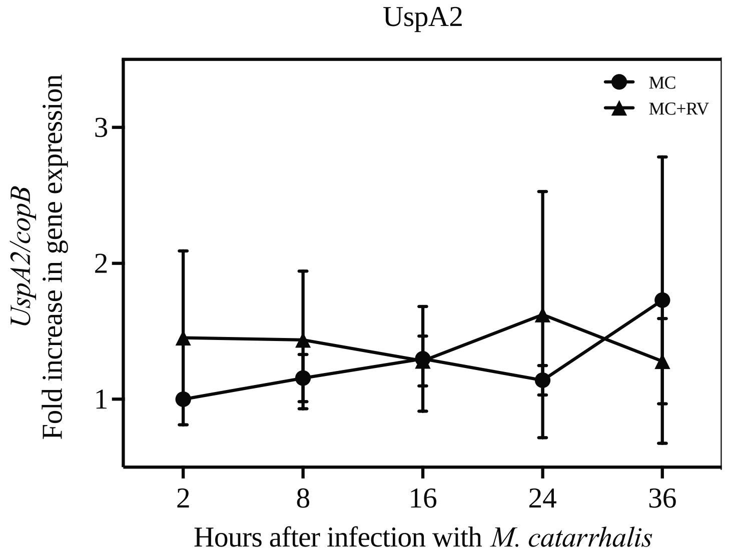

Figure S2: *UspA* expression in infected ALI cultures
Fully differentiated ALI cultures were infected with RV-C15 for 16 hours, followed by *M. catarrhalis* for 2 – 36 hours. The expression of **(A)** *UspA1* and **(B)** *UspA2* was measured by qPCR using specific primers given in table 1.
